# Development of a neural efficiency metric to assess human-exoskeleton adaptations

**DOI:** 10.3389/frobt.2025.1541963

**Published:** 2025-04-02

**Authors:** Ranjana K. Mehta, Yibo Zhu, Eric B. Weston, William S. Marras

**Affiliations:** ^1^ Department of Industrial and Systems Engineering, University of Wisconsin Madison, Madison, WI, United States; ^2^ Department of Industrial and Systems Engineering, Texas A&M University, College Station, TX, United States; ^3^ Department of Integrated Systems Engineering, The Ohio State University, Columbus, OH, United States

**Keywords:** biomechanics, fNIRS, lifting, motor adaptation, neuroergonomics, exoskeleton

## Abstract

Passive exoskeletons have been introduced to alleviate loading on the lumbar spine while increasing the wearer’s productivity. However, few studies have examined the neurocognitive effects of short-term human-exoskeleton adaptation. The objective of the study was to develop a novel neural efficiency metric to assess short-term human exoskeleton adaptation during repetitive lifting. Twelve participants (gender-balanced) performed simulated asymmetric lifting tasks for a short duration (phase: early, middle, late) with and without a passive low back exoskeleton on two separate days. Phase, exoskeleton condition, and their interaction effects on biomechanical parameters, neural activation, and the novel neural efficiency metric were examined. Peak L5/S1 superior lateral shear forces were found to be significantly lower in the exoskeleton condition than in the control condition. However, other biomechanical and neural activation measures were comparable between conditions. The temporal change of the neural efficiency metric was found to follow the motor adaptation process. Compared to the control condition, participants exhibited lower efficiency during the exoskeleton-assisted lifting condition over time. The neural efficiency metric was capable of tracking the short-term task adaptation process during a highly ambulatory exoskeleton-assisted manual handling task. The exoskeleton-assisted task was less efficient and demanded a longer adaptation period than the control condition, which may impact exoskeleton acceptance and/or intent to use.

## 1 Introduction

Physical lifting, repetitive twisting, and bending motions during manual material handling (MMH) tasks are the leading causes of low-back disorders (Waddell and Burton, 2001). With the advent of Industry 3.0, a growing trend toward process automation has employed robotic equipment to accelerate production efficiency and replace hazardous MMH tasks (MacDougall, 2014). However, complete automation of some tasks is difficult to achieve owing to their complexities (Lazzaroni et al., 2018). Therefore, semi-automated approaches, such as employing exoskeletons that can reduce biomechanical loads while retaining the flexibility of human decision-making, have gained wide attention in the manufacturing and construction industries. An exoskeleton is defined as a wearable device that augments, enables, assists, or enhances a wearer’s motion, posture, or physical performance (ASTM) and can be categorized into active (those that use actuators and motor power to move) or passive (those that use springs and dampers to aid the wearer’s movements (de Looze et al., 2016)).

The biomechanical impacts of industrial passive exoskeletons have been investigated extensively on their ability to reduce biomechanical loads on the wearers. Reductions in low back muscle activation attributable to exoskeletons during lifting are most often quantified using electromyography (EMG (Lazzaroni et al., 2018)). Alone, EMG measures low-back muscle activation but not necessarily muscle forces, given that muscle forces are modulated by force-length and force-velocity relationships (Hwang et al., 2016a). Therefore, biomechanical modeling techniques, which predict dynamic changes of biomechanical loads on the lumbar spine during the exoskeleton-assisted tasks (Picchiotti et al., 2019; Weston et al., 2018), hold promise to monitor the adaptation process over time directly and have reported biomechanical loading trade-offs.

The cognitive fit of the human-exoskeleton interaction, which ensures the wearer is adapted to the task both physically and cognitively, evolves slowly and has not been prioritized for exoskeleton designs (Stirling et al., 2020). Physical movement during initial exposure to passive exoskeletons demands the wearers to adapt to the new motor demands (Cothros et al., 2006; Gordon and Ferris, 2007). For example, previous exoskeleton-based locomotor adaptation studies reported that wearing an exoskeleton disrupts neuromuscular coordination, as evidenced by increased muscle activation to fight the rigid and resistive exoskeleton structure (Gordon and Ferris, 2007), thus requiring a longer locomotor adaptation period (Gordon and Ferris, 2007; Galle et al., 2013). With the development of advanced control algorithms, kinematic and metabolic-based exoskeletons have demonstrated superior adaptation capabilities over passive exoskeletons (Gordon et al., 2013; Panizzolo et al., 2019). However, these traditional locomotor control algorithms aim to optimize the wearers’ gait performance based on their body kinematics, EMG, and metabolic measurements (Belda-Lois et al., 2011) and ignore the neurocognitive requirements when wearers interact with exoskeletons.

An increasing number of studies have been conducted that employ neuroergonomic (i.e., study of brain and behavior at work) approaches to understand motor skill learning and adaptation outcomes based on wearers’ brain responses (Ang et al., 2009; Rea et al., 2014). In a dynamic locomotor adaptation task, the bilateral left/right dorsolateral prefrontal cortex (L/RdlPFC), known for its regulatory role in executive function, such as decision-making (Ahlstrom et al., 2016) and working memory (Ahn et al., 2016; Foy and Chapman, 2018), was constantly engaged to adapt to the changing gait speed by maintaining appropriate walking posture (Miyai et al., 2001; Suzuki et al., 2004). Motor adaptation studies using functional magnetic resonance imaging also revealed that motor planning regions, especially the right premotor cortex (RPMC), are not only involved in movement preparation but also engage with RdlPFC to support visuospatial cognitive processes during the early phases of motor-task adaptation (Eversheim and Bock, 2001; Seidler et al., 2006). Functional connectivity between the right-lateralized prefrontal and premotor cortexes was observed during lifting with passive exoskeleton use, which implied the activation of the action monitoring system (Zhu et al., 2021).

Advances in ambulatory neuroimaging technologies have opened avenues for the development of objective metrics to quantify the neurocognitive cost of preserving or maintaining ambulatory motor performance (McKendrick et al., 2017; Mehta and Parasuraman, 2013) and thus hold promise to capture motor-task adaptation with exoskeletons over time directly. Dynamic changes in neural activity have been employed to infer task adaptation and expertise development (Curtin et al., 2019a). For example, Curtin (Curtin et al., 2019b) reported increases in cognitive task performance with training over time that were associated with decreased neural activation in the dlPFC. They attributed the observed brain-behavior dynamics over time to the neural efficiency (NE) hypothesis, proposing that smart individuals are more efficient by utilizing less neural effort to achieve greater task performance (Haier et al., 1988). Several studies have utilized various advanced neuroimaging techniques to test the neural efficiency hypothesis and have reported that neural efficiency is also a function of expertise level, which can be developed through practice and task adaptation (Dunst et al., 2014; Sayala et al., 2006). In the domain of human-exoskeleton interactions, neural efficiency metrics have been previously applied to track the training progress of an exoskeleton-assisted gait rehabilitation program for both stroke and spinal cord injury patients (Zhu et al., 2020a).

It is essential that the design of industrial exoskeletons as ergonomic interventions to reduce biomechanical loading not only optimizes both the physical and cognitive fit but also promotes efficient motor-task adaptation processes (Stirling et al., 2020; Stirling et al., 2019). The objective of this study was to monitor human-exoskeleton interaction using a promising neural efficiency metric to better understand and quantify short-term motor adaptation with an industrial passive low-back exoskeleton during a repetitive asymmetric MMH task. We hypothesized that a low-back exoskeleton-assisted asymmetric lifting task is associated with poor (negative) neural efficiency at the beginning of the task, indicating greater cognitive processing and motor planning demands. Additionally, we hypothesize that the metric will improve as the wearers adapt to the exoskeleton-assisted asymmetric lifting task over time.

## 2 Methods

### 2.1 Participants

Twelve healthy young adults (6 males and 6 females) with no self-reported history of low back injuries were recruited to perform simulated asymmetric MMH tasks on separate days. The demographic data (Mean ± SD) of participants is summarized in Table 1. The experimental protocol was approved by the Ohio State University Institutional Review Board [IRB # 2018H0569], and all participants provided written consent at the start of the study.

**TABLE 1:** Participant demographics (mean ± SD).

	Male	Female	Total
n	6	6	12
Age (years)	28.8 ± 4.8	24.6 ± 4.4	26.9 ± 4.7
Height (m)	1.790 ± 0.045	1.722 ± 0.046	1.759 ± 0.053
Weight (kg)	74.7 ± 12.2	59.5 ± 13.0	67.8 ± 11.6
BMI	23.31 ± 2.64	20.06 ± 3.31	21.91 ± 2.43

### 2.2 Instrumentation

**FIGURE 1:**
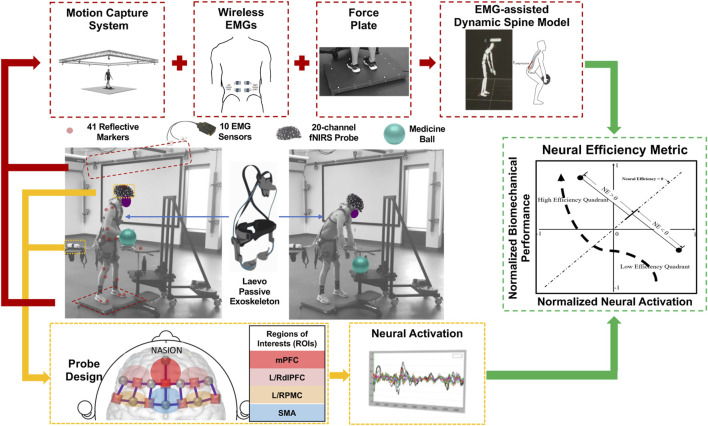
Biomechanical and neuroergonomic feature extraction process during MMH tasks. A 20-channel fNIRS system monitored the task-related physiological changes simultaneously with the aforementioned EMG-assisted spine model. Neural activation of 6 regions of interest (ROIs), namely, right and left dorsolateral prefrontal cortex (RdlPFC and LdlPFC), medial prefrontal cortex (mPFC), left/right premotor cortex (LPMC/RPMC), and supplementary motor area (SMA), were monitored. The neural efficiency metric was developed based on the output features from both systems.

The tested passive low-back exoskeleton was the LaevoTM 2.5 (Figure 1; Laevo, the Netherlands), which consists of a chest pad and two leg pads that are connected by multiple elastic beams. During an MMH task, the device harvests the kinetic energy during the lowering phase and restores the energy to aid lifting. Biomechanical loads in the lower back were measured using an established EMG-assisted dynamic spine model (Hwang et al., 2016a; Hwang et al., 2016b). The model relies on dynamic inputs, including muscle activity for the ten power-producing muscles of the trunk, full-body kinematics, and ground reaction forces. These dynamic inputs are also combined with more “static” inputs, such as anthropometry, muscle geometry, and tissue material properties. According to standardized placement procedures (Mirka and Marras, 1993), ten wireless EMG sensors with a sampling rate of 1925.93 Hz (TrignoTM, Delsys, MA, United States) were placed on bilateral trunk muscles, namely, the erector spinae, latissimus dorsi, rectus abdominis, internal oblique, and external oblique. Participants’ body kinematics data were collected via an OptiTrack Motion Capture System with a sampling frequency of 120 Hz (NaturalPoint, OR, United States) and processed through a customized Matlab-based laboratory software (Mathworks, MA, United States. Kinetic ground reaction force and moments were recorded using an FP6090-15 force plate at the frequency of 1,000 Hz (Bertec, OH, United States). The EMG and kinematics data were synchronized using a USB-6225 data acquisition board (National Instruments, TX, United States).

In this study, neural activation of each participant was monitored using a 20-channel portable continuous wave Functional Near Infrared Spectroscopy (fNIRS) system, NIRSportTM (NIRx Medical Technologies, NY, United States), which included 8 emitters (marked in circles, Figure 1) and 7 detectors (marked in squares, Figure 1). The light signals are emitted in two wavelengths (760 and 850 nm) and the probe design is represented according to the international 10–20 system format. The inter-optode distance for the 20 standard-length channels was set as 3 cm. An additional eight short separation detectors, each with an inter-optode distance of 8 mm, were placed next to each emitter. Signals from short separation channels were used to correct for physiological noise and motion artifacts during the data collection (Yücel et al., 2015). The fNIRS probe, designed in NIRSITETM (NIRx Medical Technology, NY, United States), covered 6 regions of interest (ROIs), namely, the right and left dorsolateral prefrontal cortex (RdlPFC and LdlPFC), medial prefrontal cortex (mPFC), left/right premotor cortex (LPMC/RPMC), and supplementary motor area (SMA) based on the clinically anatomy-based Brodmann areas and their international 10–20 system locations (Figure 1; (Homan et al., 1987)). Previous locomotor adaptation studies have demonstrated that motor planning-related cortical regions, i.e., SMA and PMC, are also associated with the motor learning process in able-bodied participants (Miyai et al., 2001). During exoskeleton-assisted dynamic motor tasks, increased motor control and working memory levels are also required during the initial learning phase by engaging bilateral dlPFC (Kao et al., 2010; Suzuki et al., 2004; Suzuki et al., 2008; Tyagi et al., 2023). Thus, bilateral dlPFC, mPFC, SMA, and bilateral PMC were monitored to quantify the exoskeleton-associated sensorimotor adaptation process.

### 2.3 Experimental procedures

Participants completed a two-session experiment on separate days. Before each asymmetric lifting session, anthropometric data were collected from the participants, including subject height, weight, and the width/depth/circumference of the torso at the xiphoid process and umbilicus. 10 wireless EMG sensors were fixed to the trunk muscles, and 41 reflective optical motion-capturing markers were placed on the entire body of each participant according to a custom marker set prescribed by the OptiTack motion capture software. Three markers were also placed on the force plate to track its relative location during the asymmetric lifting task. Next, the fNIRS probe was strapped to each participant with the center of the cap placed on the vertex (Cz) of the head. The probe was fully covered by a black shower cap to eliminate the signal contamination from both the ambient light and infrared light emitted from the cameras of the motion capture system. After sensor placement, the fNIRS system was first calibrated by having each participant seated upright and fully relaxed without any movement for 3 min (Zhu et al., 2020b).

After sensor calibration, the hip center of rotation and strap circumferences were fitted to participants. Participants then went through another calibration process for the dynamic spine model by conducting a series of lowering and lifting motions while holding a 20-pound medicine ball. During this step, gain ratios are reverse-engineered for each of the 10 power-producing muscles of the lumbar spine. EMG activations are then combined with this gain ratio, muscle cross-section area, length, and contraction velocity information to predict dynamic outputs of muscle force. Separate calibrations were conducted for each participant and experimental session. For familiarization, all participants were asked to practice the task for at least 5 minutes before the actual physical experiment began. The exoskeleton was adjusted, based on the manufacturer’s guidelines, to allow participants to comfortably operate the physical task.

For the experimental task, participants were instructed to lift a 16-pound medicine ball back and forth between a lift origin/destination at knee height at 45° asymmetry relative to the sagittal plane and a lift origin/destination at waist height in front of the body. Lifts were performed at a frequency of 6 lifting-and-lowering trials per minute for 30 min, paced by a programmed metronome. All participants performed the tasks with and without wearing the exoskeleton on two separate days with counterbalancing, and the two sessions were separated by a minimum of 24 h to allow for adequate rest and recovery.

### 2.4 Experimental design

A 2 × 3 repeated measures design was employed in this study to evaluate the effects of exoskeleton condition (exoskeleton vs. control), phase (early: averaged the fifth and 10th minutes; vs. middle: averaged 15th and 20th minutes; vs. late: averaged the 20th and 30th minutes), and their interaction effects on biomechanical, neural activation and neural efficiency measures. Raw biomechanical modeling and neural activation signals were first synchronized within each 1-min window with respect to the metronome pacing signal. Dependent variables, discussed in the following section, were then calculated per lifting-and-lowering trial and averaged into early, middle, and late phases for statistical analysis.

### 2.5 Measurements

EMG signals obtained from ten trunk muscles were pre-processed by notch filtering at 60 Hz and band-pass filtering between 30 and 450 Hz. Following the standards for reporting EMG results (Merletti and Di Torino, 1999), the filtered signals were rectified, smoothed, and low-pass filtered using a 2nd order Butterworth filter with a cut-off frequency of 1.59 Hz. Similarly, kinematic data were low-pass filtered using a 4^th^-order Butterworth filter at a cut-off frequency of 10 Hz. After passing dynamic inputs (EMG, kinematics, kinetics) and “static” inputs (subject anthropometry, Magnetic Resonance Imaging (MRI)-derived muscle sizes and locations, tissue material properties) into the multi-body dynamic solver (Adams, MSC Software, Santa Ana, CA, United States), dynamic, three-dimensional spinal loads (compression, anterior/posterior or A/P shear, lateral shear) on the superior and inferior endplates extending from the T12/L1 to L5/S1 were derived as model outputs (Hwang et al., 2016b). Peak compression, anterior/posterior (A/P) shear, lateral shear, and resultant spinal loads were extracted for the vertebral endplate, for which the highest magnitudes of loading were observed along each spinal loading dimension during every lifting-and-lowering trial for further analysis (Picchiotti et al., 2019). These peak loads occurred at L3/L4 (for compression and resultant spinal loading) and L5/S1 (for A/P and lateral shear loading).

**FIGURE 2:**
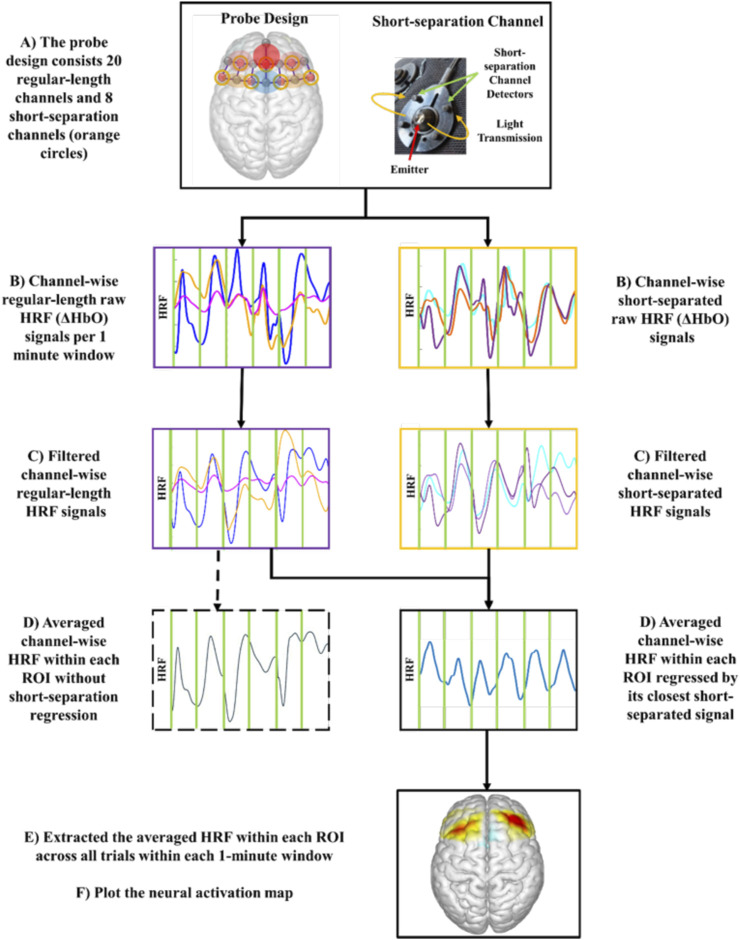
Experimental fNIRS signal processing flow chart to generate neural activation map.

Acquired fNIRS raw data were pre-processed using the NIRS Brain AnalyzIR toolbox (Figure 2; (Santosa et al., 2018)). The collected light intensity signals were first converted to optical densities by taking logarithms. To reduce the effect of physiological noises such as cardiovascular pulsations and Mayer Waves (Julien, 2006), the converted optical density signals were first band-pass filtered between 0.01 and 0.4 Hz and then subjected to principal component analysis (PCA) among all regular length channel signals (Cao et al., 2015; Zhang et al., 2005). A combination of spline interpolation function with parameter p = 0.99 and a Kurtosis-based wavelet transformation function with kurtosis = 3.3 was applied to minimize the effect of motion artifacts (Chiarelli et al., 2015; Scholkmann et al., 2010).

The hemodynamic response functions (HRF) of oxyhemoglobin (ΔHbO) concentration were obtained using the modified Beer-Lambert law (Delpy et al., 1988). HRF of ΔHbO was selected for neural activation analysis due to its strong correlation with motor task-related brain activation compared to HRF of deoxyhemoglobin (ΔHbR) signal (Malonek and Grinvald, 1996). To further reduce the effect of physiological noise and task-related motional artifacts during the highly ambulatory MMH tasks, the obtained HRFs were fed into a General Linear Model (GLM) using the Iteratively Reweight Least-Square Autoregressive pre-whitening approach (AR-IRLS; (Santosa et al., 2018)), where the HRF of each short-separation channel served as a regressor for its closest standard-length channels. After exporting the estimated HRF of each regular-length channel, the task-related neural activation was obtained by extracting the averaged channel-wise HRF within each ROI in every lifting-and-lowering trial (von Lühmann et al., 2020).

To compute the neural efficiency (NE) of each lifting-and-lowering trial, the biomechanical performance parameters and neural activations within each ROI were first normalized by converting to z scores across both experimental sessions (Figure 1). We investigated the neural efficiency changes for the biomechanical parameters over each ROI using Equation 1 below:

$${NE}_{ij}=\frac{z\left(p_{i}\right)-z\left(e_{j}\right)}{\sqrt{2}}$$ (1)

Where i ∈ {L3/L4 Inferior Compression, L5/S1 Inferior A/P Shear, and L5/S1 Superior Lateral Shear, and L3/L4 Resultant}; j = {RdlPFC, and RPMC}, p_i_ is the *i*th biomechanical performance parameter and e_j_ is the oxygenated hemoglobin level in the *j*th ROI. During the passive low back exoskeleton-assisted asymmetric lifting task, wearers were expected to achieve higher biomechanical performance (i.e., lower spinal loading) with decreased neural activation of the monitored ROIs, from the low-efficiency quadrant (the fourth quadrant of the Neural Efficiency Metric graph in Figure 1) to the high-efficiency quadrant (the second quadrant of the Neural Efficiency Metric graph in Figure 1), over time. Low neural efficiency (NE < 0) was expected at the beginning of each asymmetric lifting session, which characterizes increased neural activation with decreased biomechanical performance (i.e., increased spinal loading). To achieve high neural efficiency (NE > 0) and adaptation to the asymmetric lifting task over time, wearers needed to not only enhance the biomechanical performance (i.e., decreased spinal loading) but also exhibit reduced neural activation in the motor adaptation-related regions of the brain, namely, RdlPFC, and RPMC, by adapting to the asymmetric lifting task.

### 2.6 Statistical analysis

The dependent variables, namely, the biomechanical performance parameters (namely, L3/L4 Inferior Compression, L5/S1 Inferior A/P Shear, and L5/S1 Superior Lateral Shear, and L3/L4 Resultant), neural activation of all ROIs, and RdlPFC and RPMC related neural efficiency metrics, were calculated per lifting-and-lowering trial and averaged into early, middle, and late phases for statistical analysis. Separate two-way repeated measure analyses of variance (ANOVAs) were conducted to test the main effect of phase (early vs. middle vs. late), condition (exoskeleton vs. control), and their interaction on the biomechanical performance parameters (namely, L3/L4 Inferior Compression, L5/S1 Inferior A/P Shear, and L5/S1 Superior Lateral Shear, and L3/L4 Resultant), neural activation of all ROIs, and RdlPFC and RPMC related neural efficiency metrics separately. Yeo-Johnson transformation was applied if the dataset violated the assumption of normality using the PowerTransformer data preprocessing toolbox of the scikit-learn package (Yeo and Johnson, 2000). Statistical significance was tested with alpha = 0.05, and False Discovery Rate (FDR) correction (with a desired significance level of q = 0.050) was applied to account for multiple comparisons.

## 3 Results

### 3.1 Biomechanical performance

**FIGURE 3:**
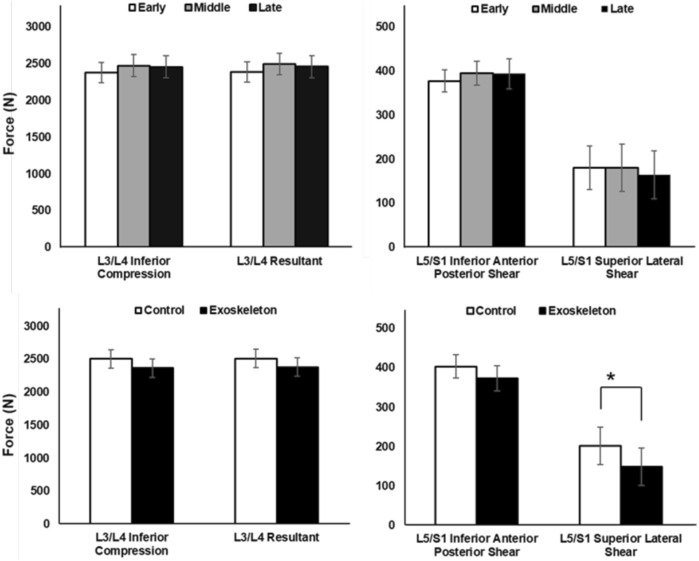
Peak spinal compressive and shear forces across phase and condition effects, averaged across participants (Mean ± SD). No significant phase (top) and condition (bottom) main effects were observed for both compression (left) and shear (right) forces on the lumbar spine, except L5/S1 Superior Lateral Shear, which showed significantly greater shear force in the control condition than exoskeleton condition (*p = 0.044).

Peak biomechanical loads on the L3/L4 and L5/S1 levels of the lumbar spine are illustrated in Figure 3 across phases and conditions. In general, no significant condition, phase, or condition × phase interaction effects were observed for L3/L4 Inferior Compression, L5/S1 Inferior Anterior Posterior Shear, or L3/L4 Resultant loading, with all the p-values >0.05. However, peak L5/S1 Superior Lateral Shear was found to be significantly lower in the exoskeleton condition than the control condition [F (1,11) = 5.328, p = 0.044, η_p_^2^ = 0.348].

**FIGURE 4:**
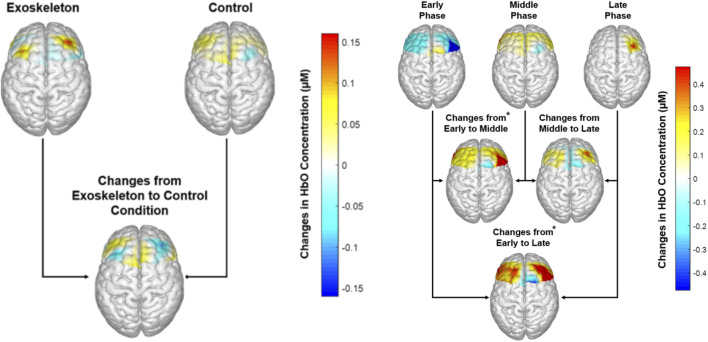
Left: Neural activation across each condition, averaged across participants. No significant condition main effect was observed (p-values ≥0.24); Right: Neural activation across each phase, averaged across participants. * Indicates higher neural activation in the RdlPFC and RPMC regions in the Middle (p values ≤0.006) and Late Phases (p values ≤0.015) compared to the Early phase.

Neural activation Figure 4 (left) illustrates the contrast maps of neural activation in each condition. No significant condition or condition × phase interaction effects were observed in the neural activation across all ROIs, with all the FDR-adjusted p-values >0.24. However, significant phase main effects were identified for the neural activation in the RdlPFC [F (1,11) = 9.454, p = 0.015, η_p_^2^ = 0.654; Figure 4 (right)] and RPMC [F (1,11) = 8.337, p = 0.006, η_p_^2^ = 0.625; Figure 4 (right)] regions. Post hoc analysis revealed that for both ROIs, neural activation in the late (p values ≤0.015) and middle (p values ≤0.016) phases was significantly greater than that in the early phase.

### 3.2 Neural efficiency

**FIGURE 5:**
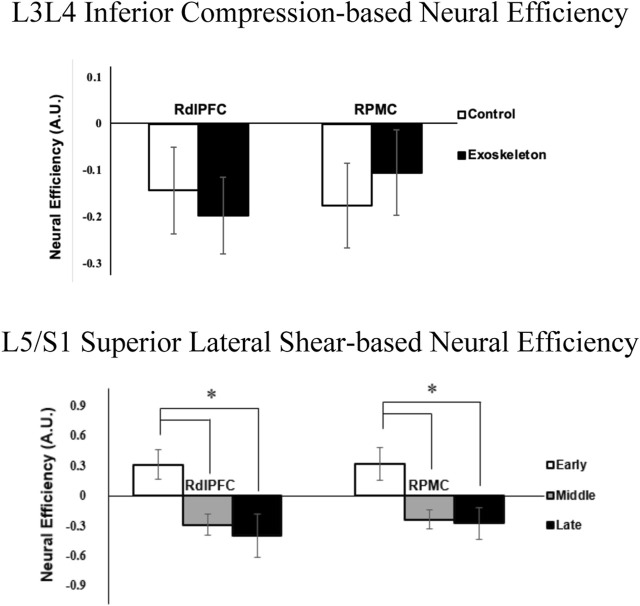
Neural efficiency metrics (averaged across participants) associated with L3/L4 Inferior Compression and RdlPFC (p = 0.059; η_p_^2^ = 0.323) between exoskeleton and control conditions (top), and L5/S1 Superior Lateral Shear with RdlPFC (*represent p = 0.048) and RPMC (*represent p = 0.024) regions over phases (bottom). Error bars are ± standard errors.

The neural efficiency metric of the L3L4 Inferior Compression [F (1,11) = 5.253, p = 0.059, η_p_^2^ = 0.323; Figure 5(top)] in the RdlPFC region, while not statistically significant, was lower in the exoskeleton condition compared to the control condition. Additionally, significant phase main effects were identified for the L5/S1 Superior Lateral Shear-related neural efficiency metric in the RdlPFC [F (1,11) = 8.367, p = 0.048, η_p_^2^ = 0.432; Figure 5 (bottom)] and the RPMC [F (1,11) = 8.319, p = 0.024, η_p_^2^ = 0.431; Figure 5 (bottom)]. In both cases, neural efficiency in the early phases was found to be significantly greater than in the middle and late phases (p values ≤0.009). No other condition, phase, or interaction effects were observed for the neural efficiency metrics (all p’s > 0.067).

**FIGURE 6:**
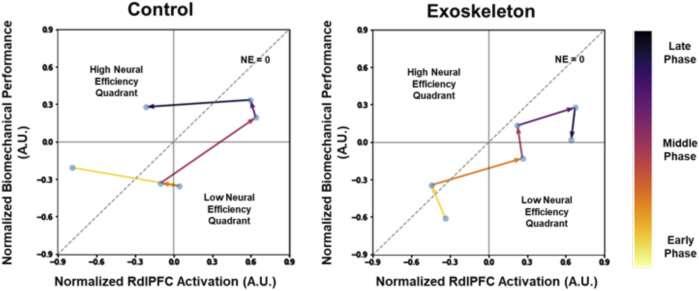
Dynamic neural efficiency maps of the L3/L4 Inferior Compression in RdlPFC. The data represents averaged values across participants. The lower the compressive loads on the spine, the greater the biomechanical performance. Note that low-efficiency quadrants are represented by lower biomechanical performance, but higher neural activation and high-efficiency quadrants are represented by higher biomechanical performance and lower neural activation.

Figure 6 illustrates the changes in the neural efficiency patterns over time (averaged across participants) at each data collected time point, namely, the L3/L4 Inferior Compression in RdlPFC. Note that the neural efficiency was lower in the exoskeleton than in the control condition when averaged across the 30 min (see Figure 5). However, when tracking the neural efficiency metric every 5 minutes, we observe different patterns in the control and the exoskeleton conditions over time.

## 4 Discussion

Here, a novel neural efficiency metric, which links the wearer’s biomechanical performance and associated neurocognitive effort, was utilized to track human-exoskeleton interaction over time.

Exoskeleton-assisted locomotor training literature has emphasized that wearing an exoskeleton hinders neuromuscular coordination and leads to poor gait performance, especially during the early phase of the training session; however, underlying neural mechanisms were not studied (Gordon and Ferris, 2007; Gordon et al., 2013; Panizzolo et al., 2019). In the present study, neural efficiency, which combines wearers’ biomechanical performance and the neurocognitive effort to maintain the performance, was captured for the first time during an exoskeleton-assisted ambulatory lifting task. In general, we found that the passive low-back exoskeleton-assisted asymmetric lifting task was associated with poorer compressive loads-right dorsolateral prefrontal cortex (RdlPFC) related efficiency than the control condition (Figure 5). While not statistically significant, this effect was accompanied with a large effect size (η_p_^2^ = 0.323). The observed inefficiency was likely a result of the increase in neurocognitive demand (Figure 3) in the RdlPFC to maintain the biomechanical performance, i.e., the compressive load, which were found to be comparable between two conditions (Figure 3 Bottom left). Bilateral dlPFC is known for its regulation of executive functions (Banich, 2009; Sibi et al., 2016). In particular, engagement of the RdlPFC, a key cortical region that is responsible for sensorimotor adaptation associated with working memory (Anguera et al., 2011; Ruitenberg et al., 2018), indicated that short-term exoskeleton interaction required a sensorimotor adaptation process for the lifting task in our study. In a recent study, Seidler, Gluskin, and Greeley (Seidler et al., 2017) applied anodal transcranial Direct-Current stimulation to boost the left or right prefrontal or motor cortex activities during a multi-session dart-throwing task. The authors reported an accelerated motor-task adaptation process with increased engagement of the right dorsolateral prefrontal cortex (RdlPFC), which validates the critical role RdlPFC plays in the motor-task adaptation process. These studies highlighted the importance of RdlPFC in motor task adaptation processes. We found that the tested passive low-back exoskeleton-assisted asymmetric lifting task is associated with lower RdlPFC neural efficiency as the exoskeleton demands greater adaptation-associated cognitive processing effort from the wearers.

Sensorimotor adaptation is an error-driven movement calibration process (Ruitenberg et al., 2018; Bastian, 2008; Seidler et al., 2012). Functional neuroimaging studies have revealed that the bilateral premotor cortices play a crucial role in the motor adaptation process, which involves movement selection, planning, calibration, and execution (Lee and van Donkelaar, 2006; Peck et al., 2009). During complex motor tasks, the prefrontal cortex is known to co-activate with premotor cortices and supplementary motor areas and functions as the central executive system to support motor planning and execution (Nee and D’Esposito, 2016). Specifically, the dorsolateral premotor-prefrontal cortical complex (PMC-dlPFC) is in charge of forming and selecting motor tasks that drive movement (Soltani and Koechlin, 2022). In this study, we observed decreased neural efficiencies of both RdlPFC and RPMC in the lumbar shear loads from early to the middle and late phases of the MMH task, suggesting that the asymmetrical lifting task efficiency declined over time irrespective of the exoskeleton condition (Figure 5). Our findings are consistent with prior literature that report that right-lateralized activation in prefrontal and premotor regions is associated with one’s spatial cognitive processing effort during the process of kinematic motor adaptation (Seidler et al., 2006). When adapting to a new motor task, increased spatial cognitive processing effort is required in an attempt to minimize the sensory prediction error, i.e., the difference between the brain’s predicted body movement and the observed body movement, by calibrating body dynamics continuously through repetitive physical practice. As such, our results suggest that the asymmetrical lifting task demands greater cognitive control and movement calibration efforts.

The dynamic increase in neural efficiency of the bilateral prefrontal cortex has been identified as a strong indicator of cognitive (Curtin et al., 2019b) and motor (Karim et al., 2017) task learning and adaptation process. In this study, dynamic changes in key neural efficiency metrics implicated exoskeleton-related differences in task adaptation processes. First, declines in efficiency from the early to the middle phases were observed in both exoskeleton and control conditions (Figure 6). The results suggested the participants struggled with their initial exposure to the asymmetric lifting task, evidenced by the decreased biomechanical performance and increased neurocognitive effort. Over time, neural efficiencies returned to the high-efficiency quadrant in the control condition, indicating that the participants were able to adapt to the lifting task by the end of the session (Figure 6 left). However, further declines in neural efficiencies during the exoskeleton condition suggest that the tested passive low-back exoskeleton did not promote an efficient lifting task adaptation process, as observed in the control condition, and that potentially longer adaptation periods are needed (Figure 6 right). These findings do not necessarily place exoskeleton use in a poor light but instead implicate the importance of worker training strategies that are needed for effective and fluent human-exoskeleton interactions. Indeed, a recent study reported different adaptation strategies between shoulder exoskeleton and control groups when performing dual tasks over days (Tyagi et al., 2023).

In this study, only short-term adaptation (∼30 min) with the exoskeleton was explored; however, repetitive long-term motor-task adaptation is important to fully master motor skills (Ruitenberg et al., 2018), (Bastian, 2008). As such, future studies that examine long-term adaptation and/or re-adaptation processes on subsequent days are warranted to provide a comprehensive understanding of human-exoskeleton interactions and to facilitate the development of adaptive training programs (Mehta et al., 2022). Additionally, because the user experience with MMH tasks, with or without exoskeleton use, may also impact the motor adaptation process, future work should assess how expertise impacts the neural efficiency metric outcomes. It is also possible that different individuals prescribe different movement strategies, and thus, individual variability may impact the group-averaged results reported here. Future work that adopts individual-specific analyses with larger sample sizes to assess the utility of the neural efficiency metric is warranted. The neural efficiency metric has the potential to evaluate efficiency profiles in a variety of users for immediate or short-term experiences with various exoskeleton designs/models. Therefore, a systematic combination of exoskeleton designs and motor task scenarios should be tested using the neural efficiency metric. Such an investigation will enable the metric’s generalizability and utility towards 1) comprehensive evaluation of exoskeleton designs during the design life cycle; 2) determination of exoskeleton usage on a variety of tasks; and 3) development of training programs that promote efficient human-exoskeleton adaptation. Surprisingly, spinal loads were not associated with the motor adaptation process in this study, likely due to the tested low-intensity MMH task condition (compared to the established lumbar biomechanical load limits: >3400 N compressive load, >700 N shear force (Gallagher and Marras, 2012; American Conference of Governmental Industrial Hygienists, 2001)). Thus, future studies should also investigate human-exoskeleton interactions associated with the motor adaptation process for more physically demanding tasks (e.g., high load, long duration tasks) that the passive low-back exoskeletons are designed for.

## 5 Conclusion

In the present study, traditional physical ergonomic and neuroimaging metrics failed to identify significant differences in human exoskeleton adaptations independently. The neural efficiency metric, which incorporates these traditional metrics, indicated that the exoskeleton-assisted task was less efficient and demanded a longer adaptation period than the control condition. The proposed neuroergonomic evaluation metric may be applied during formative user testing, which may inform hardware technologists of the impact of different exoskeleton design parameters on the wearers’ neuromotor task adaptation under various task scenarios. This study also lays the groundwork for future research that can facilitate the development of technical standards and guidelines on exoskeleton use and training needs based on exoskeleton-assisted task efficiency.

## Data Availability

The raw data supporting the conclusions of this article will be made available by the authors, without undue reservation.
